# Alleviation of cadmium and drought stress in wheat by improving growth and chlorophyll contents amended with GA3 enriched deashed biochar

**DOI:** 10.1038/s41598-023-45670-7

**Published:** 2023-10-28

**Authors:** Tauseef Anwar, Asma Shehzadi, Huma Qureshi, Muhammad Nadeem Shah, Subhan Danish, Saleh H. Salmen, Mohammad Javed Ansari

**Affiliations:** 1 Department of Botany, The Islamia University of Bahawalpur, Bahawalpur, Pakistan; 2 Department of Botany, University of Chakwal, Chakwal, Pakistan; 3 Department of Agriculture, Government College University, Lahore, Pakistan; 4 North Florida Research and Education Center, University of Florida, 155 Research Road, Quincy, FL USA; 5 Department of Soil Science, Faculty of Agricultural Sciences and Technology, Bahauddin Zakariya University, Multan, Pakistan; 6 Department of Botany and Microbiology, College of Science, King Saud University, PO Box-2455, 11451 Riyadh, Saudi Arabia; 7 Department of Botany, Hindu College Moradabad (Mahatma Jyotiba Phule Rohilkhand University Bareilly), Moradabad, 244001 India

**Keywords:** Plant sciences, Plant stress responses, Abiotic, Drought

## Abstract

Drought and cadmium (Cd) stress are both major issues that significantly affect the growth and development of wheat plants. Both drought stress and Cd toxicity disrupt physiological processes i.e., nutrient uptake, cell expansion, and enzymatic reactions resulting in poor crop growth. To overcome these issues, the use of activated carbon and gibberellic acid (GA3) are considered valuable amendments. However, the current study aimed to add value using GA3-enriched biochar (GA3-BC). That’s why, a lab experiment was conducted on wheat to assess the effectiveness of GA3-BC against Cd and drought stress. For GA3 enrichment in biochar, 10 µg GA3/g biochar was mixed. There were 3 levels of GA3-BC i.e., 0, 0.6 (GA3-BC1), and 0.9% (GA3-BC). All levels were applied in 3 replicates under no stress (0Cd + no drought), drought stress (DS), and 6 mg Cd/ kg soil (6Cd). Results showed that GA3-BC2 caused a significant improvement in shoot length (44.99%), root length (99.73%), seedling length (60.13%) and shoot fresh weight (63.59%) over control at 6Cd + drought stress. A significant improvement in chlorophyll a, chlorophyll b, and total chlorophyll while a decrease in electrolyte leakage and regulation of antioxidants i.e., lipid peroxidation, SOD, CAT, APx, GR, GPx, GST, and DPHH also signified the effectiveness of GA3-BC2 compared to control at 6Cd + drought stress. In conclusion, GA3-BC2 is an efficacious amendment for simultaneously alleviating drought and Cd stress in wheat. More investigations are recommended at the field level on different cereal crops cultivated in different soil textures to declare GA3-BC2 as the best treatment for mitigation of drought stress and Cd toxicity.

## Introduction

Drought stress is a significant environmental factor that poses a significant challenge to plant growth, development, and productivity^1,2^. It remains a critical concern for agricultural researchers and plant breeders alike. Disturbingly, projections indicate that by 2025, approximately 1.8 billion individuals will encounter severe water scarcity, with 65% of the global population residing in water-stressed environments^3^. Drought stress occurs when plants experience a prolonged period of inadequate water availability due to limited rainfall, high temperatures, or soil water deficit. It triggers a cascade of physiological, biochemical, and molecular responses in plants, leading to various detrimental effects^4^. When drought stress occurs, water deficiency can cause cellular dehydration, disruptingell membrane integrity^5^. This makes the membranes more permeable, allowing electrolytes to escape from the cells. This increased electrolytic leakage is an indicator of cellular damage and membrane integrity loss^6,7^.

On the other hand, soil serves as a natural habitat, but it can face contamination from the accumulation of heavy metals (HMs), which are metals and metalloids with densities exceeding 5 g cm^−3^^8^. Among different heavy metals, cadmium (Cd) is considered the most notorious due to its high solubility in water^9,10^. Higher uptake of Cd can have a detrimental effect on the process of photosynthesis. This can be seen in the many physiological changes that occur in the plant, such as the accumulation of metals in foliage, changes in chloroplast membrane function, alterations in leaf tissue composition, a decrease in the formation of photosynthetic pigments, modifications in cytoplasmic enzymes and organics, and the destruction of enzymes related to photosynthetic carbon reduction and the xanthophyll cycle^11^. Cadmium in particular, stunted growth due to the oxidative damage^12^. This oxidative stress prevents the synthesis of enzymes responsible for producing chlorophyll^13^.

Activated carbon (biochar) is considered one of the most popular amendments to overcome these critical issues. Biochar is a carbon-rich material obtained through the pyrolysis of organic matter, such as agricultural waste, wood chips, or plant residues^14–16^. It is known for improving soil fertility, enhancing nutrient retention, and promotingicrobial activity^17–19^. It can increase water-holding capacity in the soil, enhance nutrient availability to plants, and improve soil structure, ultimately leading to improved plant growth and productivity^20^. However, the impact of deashed biochar still needs significant attention for its potential use as an amendment against drought stress and Cd toxicity.

Furthermore, gibberellic acid (GA3) is a plant growth regulator crucial to various physiological processes. GA3 is involved in seed germination, stem elongation, flowering, and fruit development. It can promote cell division and elongation, stimulate enzyme activity, and regulate gene expression related to plant growth and development^21^. Cruz-Castillo et al. demonstrated that the application of gibberellic acid (GA3) to the pedicel of Hayward kiwifruit resulted in the elongation of the terminal pedicel and had an impact on the fruit's shape index^22^. Similarly, Liu et al. reported a significant increase in apple fruit stalk length and improved fruit shape following GA3 treatment^23^. In another study involving Chandler strawberries, it was observed that the combined application of GA3 and cytokinin, substances known for their cell division activity, promoted cell elongation, leading to an increase in fruit stalk length and an improved fruit shape index^24^. Notably, GA3 treatment in tomatoes has been shown to stimulate cell expansion and enlargement, ultimately enhancing the fruit's shape index^25^. Moreover, the application of GA3 via spraying on self-pollinated apple plants led to increased fruit weight, a reduction in the proportion of asymmetric fruits, and the restoration of external fruit shape and quality to levels comparable to cross-pollinated fruits^23^.

Wheat is of paramount importance as a staple food due to its role in providing essential nutrients, versatility in culinary applications, economic significance, and global availability. It serves as a primary source of carbohydrates, dietary fiber, protein, vitamins, and minerals, making it a fundamental dietary component for billions^26,27^. Keeping in mind the importance of wheat as a nutritive and staple food, this study addresses the pressing need to mitigate the adverse effects of cadmium contamination and drought stress on wheat. We propose an innovative approach involving the use of GA3-enriched deashed biochar, which has not been explored in depth previously. The study aimed to explore the potential impact of GA3-enriched deashed biochar and its best application levels for wheat under drought stress and Cd toxicity. This study is filling the knowledge gap in using GA3-enriched deashed biochar as a management strategy for sustainable solutions to the challenges posed by cadmium pollution and drought. It is hypothesized that GA3-enriched deashed biochar has the potential to alleviate both drought and cadmium stress in wheat via the regulation of antioxidants.

## Material and methods

### Soil sampling and characterization of soil

Soil samples were collected from 0 to 15 cm depth^28^. The soil sampling site was characterized as calcareous sandy soil with low organic matter content. In the past, the soil sampling area (Research Area, Department of Botany, Islamia University Bahawalpur, Punjab, Pakistan) was utilized to produce crops. The pH of the soil-saturated paste was determined using a pH meter based on the method described by^30^. The soil organic matter (OM) was estimated using the potassium dichromate method according to^31^. The electrical conductivity (EC) of the soil was analyzed by mixing 1 part soil with 10 parts deionized water and measuring the EC using an EC meter based on method^32^. Soil phosphorus (P) was analyzed using the Olsen method described by^33^, and soil potassium (K) was analyzed using flame photometry after extracting the soil with ammonium acetate according to^34,35^.

### Seeds collection and seeds sterilization

Arooj 2022 wheat seeds were obtained from a local market for the experiment. To ensure sterility, the seeds were sterilized using a 5% sodium hypochlorite solution, followed by three rinses with 95% ethanol. The sterilization procedure involved immersing the seeds in the sodium hypochlorite solution for a duration of 30 min, followed by thorough washing with 95% ethanol three times^36^.

### Biochar

Fruits and vegetable waste from a local market situated at 30°11′29.8N 71°28′48.8 E were gathered to make biochar. Initially, the trash was dried in the sun and then chopped into small pieces. Pyrolysis was done in an aerated setting at a temperature of 325 ± 5 °C^37^, and the physical and chemical characteristics of the biochar created during the pre-experimental phase are recorded in Table 1.

**Table 1:** Pre-experimental soil, biochar, and irrigation characteristics.

Soil	Values	Biochar	Values	Irrigation	Values
pH	8.26	pH	8.21	pH	6.94
EC*e* (dS/m)	3.11	EC*e* (dS/m)	3.05	EC (µS/cm)	471
Soil Organic Matter (%)	0.65	Volatile Matter (%)	25	Carbonates (meq./L)	0.00
TN (%)	0.04	Fixed carbon (%)	45	Bicarbonates (meq./L)	4.11
EP (mg/kg)	9.87	TN (%)	0.07	Chloride (meq./L)	0.10
AK (mg/kg)	139	TP (%)	0.19	Ca + Mg (meq./L)	2.99
Sand (%)	25	TK (%)	0.33	Sodium (mg/L)	123
Silt (%)	40	TCd (µg/g)	0.09	*TN* = Total nitrogen *EP* = Extractable phosphorus *AK* = Available potassium *CEC* = Cation exchange capacity *EC* = Electrical conductivity	
Clay (%)	35	TCd = Total cadmium			
Texture	Clay loam	Particle size	< 2 mm		

### Deashing of biochar

To prepare the biochar, it was first washed with tap water to eliminate any impurities. Once the ash content was eliminated, the biochar underwent a thorough rinsing with deionized water to ensure the removal of any remaining ash residues. Subsequently, the biochar was air-dried in a well-ventilated space until it reached complete dryness^38^. Finally, the deashed biochar was properly stored for further use.

### Experimental design

The experimental design was completely randomized design. There were 2 levels of Cd i.e., control (0Cd) and 6 mg Cd/kg soil^39,40^. Furthermore, for drought stress 2 levels were maintained (no drought stress [no DS] = 65% field capacity and drought stress [DS] 35% field capacity)^41^. Gibberellic Acid 3 (GA3), with a purity of 90%, was mixed in deashed biochar in powdered form. To initiate the process, the prepared deashed biochar was grinded to get fine and homogeneous powder. After, 10 µg/g GA3 was weigh precisely on analytical balance and mixed in the biochar. Finally, this powder was used soon after preparation as amendment in the soil. As per the treatment plan i.e., 0.6 and 0.9% biochar were applied in the soil manually. After mixing biochar in soil pot filling was done. Cadmium nitrate tetrahydrate was used to introduce Cd toxicity. The detail of the salt includes Sigma-Aldrich, Product Number: 642045, Batch Number: MKCT3996, Brand: ALDRICH, CAS Number: 10022–68-1, MDL Number: MFCD00149626. For the introduction of Cd toxicity, 35 kg of soil was spiked for 21 days using cadmium nitrate. The spiking procedure involved placing the 35 kg soil in a plastic container, where we gradually added the cadmium nitrate solution while diligently mixing to ensure even distribution. Following spiking, the soil was covered to prevent contamination, and we allowed it to incubate for 21 days. The choice of this incubation period was made to allow for the interaction between cadmium and the soil matrix over an extended period, simulating real-world conditions more accurately. While making the final 6 mf Cd/kg soil concentration, the initially present 0.105 mg Cd /kg soil was also considered.

### Details of treatment

All the treatments were applied in three replicates. The treatments include T1: Control (No Cd + no DS), T2: 6mgCd/kg soil (6Cd), T3: 35% field capacity irrigation (drought/stress), T4: 6Cd + DS, T5: GA3-BC1 (GA3-biochar 0.6% + no Cd + noDS), T6: GA3-BC1 + 6Cd, T7: GA3-BC1 + DS, T8: GA3-BC1 + 6Cd + DS, T9: GA3-BC2 (GA3-biochar 0.9% + no Cd + noDS), T10: GA3-BC2 + 6Cd, T11: GA3-BC2 + DS and T12: GA3-BC2 + 6Cd + DS.

### Pots

The pots were made from a plastic bag. The dimensions of each bag were 30 cm deep and 20 cm in diameter. In each bag, 5 kg of soil was filled.

### Fertilizer application

To fulfill the macronutrient requirements of nitrogen (N), phosphorus (P), and potassium (K) in a specific area, fertilizers can be added at the recommended dose. In this case, the recommended dose is 120 kg/ha of nitrogen (0.3 g N per 5 kg soil), 90 kg/ha of phosphorus (0.225 g P per 5 kg soil), and 60 kg/ha of potassium (0.15 g K per 5 kg soil).

### Seeds collection and sowing

The wheat (Akbar 2019) seeds were purchased from the government of Punjab, Pakistan, authorized seed trader. Broken and weak seeds were isolated from healthy seeds manually. The sowing of seeds was done on 15 November 2022. In each pot, 15 seeds were sown. After germination, 5 healthy seedlings were maintained by thinning.

### Harvesting

The plants were collected 21 days after being sown. To determine their shoot and root lengths, a standard scale was used. The fresh weight of the shoot and root was measured using an analytical grade balance. To obtain the dry weight, the samples were placed in an oven and dried for 48 h at a temperature of 65 ± 5 °C.

### Chlorophyll contents

To extract the samples, they were ground in a mortar and pestle using 80% acetone. The mixture was then filtered, and the resulting solution was centrifuged to eliminate any insoluble particles. The supernatant was carefully transferred to a fresh test tube. Subsequently, the concentration of chlorophyll in the extract was determined using a spectrophotometer at two specific wavelengths, namely 663 nm and 645 nm^42^.

$${\text{Chlorophyll }}\;{\text{a }}\left( {\frac{{{\text{mg}}}}{{\text{g}}}} \right) = \frac{{\left( {12.7{ } \times {\text{ A}}663} \right){-}{ }\left( {2.69{ } \times {\text{ A}}645} \right) \times {\text{V}}}}{{1000{ } \times {\text{W}}}}$$

$${\text{Chlorophyll }}\;{\text{b }}\left( {\frac{{{\text{mg}}}}{{\text{g}}}} \right) = \frac{{\left( {22.9{ } \times {\text{ A}}645} \right){-}{ }\left( {4.68{ } \times {\text{ A}}645} \right) \times {\text{V}}}}{{1000{ } \times {\text{W}}}}$$

$${\text{Total }}\;{\text{Chlorophyll }}\left( {\frac{{{\text{mg}}}}{{\text{g}}}} \right) = { }20.2\left( {{\text{OD }}645} \right) + 8.02\left( {{\text{OD }}663} \right) \times {\text{V}}/1000{ }\left( {\text{W}} \right){ }{\text{.}}$$

### Electrolyte leakage

For each plant, three leaves were chosen randomly, and the midrib of each leaf was carefully incised using a sharp blade. The leaves were then gently washed with deionized water and dried using a paper towel. The weight of each leaf was recorded as its dry weight before submerging it in 10 mL of deionized water. The leaf-containing tubes were placed in a shaking incubator at a temperature of 25 °C for a period of two hours to attain equilibrium. The solution's initial electrical conductivity (C1) was measured using a conductivity meter. Subsequently, the leaf samples were subjected to heat in a water bath at 121 °C for 20 min to induce cell death and facilitate the release of electrolytes. After cooling the samples to room temperature, the solution's final electrical conductivity (C2)as measured^43^.

$$ \text{Electrolyte Leakage }\left( \text{\%} \right) = { }\left( {\text{C2 } - \text{ C1} }\right){ }/ \text{C1 } \times { }100$$

### Statistical Analysis

The collected data were subjected to average statistical analysis^44^. The paired comparison was performed using OriginPro 2021 software^45^, and statistical significance was determined at a significance level of *p* ≤ 0.05. To assess the correlation among the studied attributes, Principal Component Analysis (PCA) was conducted using OriginPro 2021 software.

### Ethics approval and consent to participate

We all declare that manuscript reporting studies do not involve any human participants, human data, or human tissue. So, it is not applicable.

## Results

**Figure 1:**
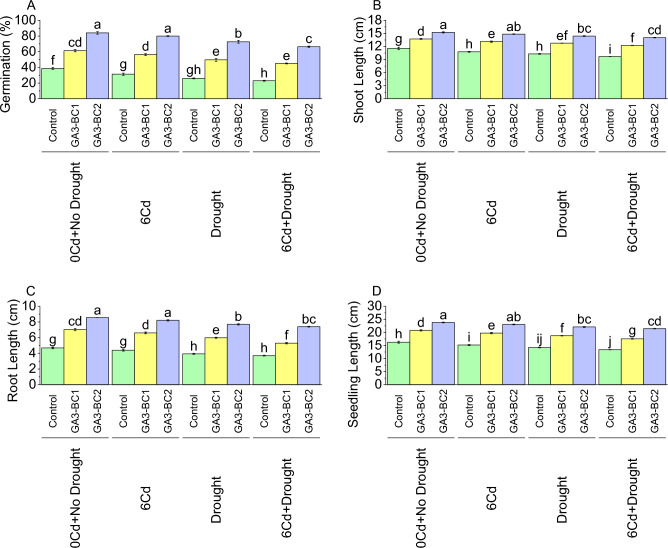
Impact of cadmium and drought stress on germination (**A**), shoot length (**B**), root length (**C**), and seedling length (**D**) of wheat amended with different levels of the GA3 enriched deashed biochar. The bars showed an average of three replicates, with standard error (SE). Statistical analysis using Tukey HSD revealed significant differences (*p* ≤ 0.05) between treatments, as indicated using different letterings on the bars.

Results showed that GA3-BC1 treatment application in 0Cd + no drought caused a significant increase (58.62%) in germination over the control (no GA3 and no BC) (Fig. 1A). Similarly, GA3-BC1 showed 79.79% increase in germination over control at 6Cd. In the case of drought stress (DS), GA3-BC1 caused a 91.03% increase compared to the control. Furthermore, when both 6Cd and DS were applied along with GA3-BC1 treatment, the germination was 45%, demonstrating an increase of 95.65% over the control. Likewise, the germination rate was significantly increased to 84%, indicating an increase (117.24%) over the control where GA3-BC2 was applied under 0Cd + no drought. It was noted that GA3-BC2 under 6Cd stress caused an enhancement in germination (155.32%) over control. Under DS, GA3-BC2 treatment resulted in 179.49% increase compared to the control. Moreover, 6Cd + DS addition of GA3-BC2 showed a 188.41% increase in germination from control.

Compared to 0Cd + no drought, 6Cd stress resulted in a 6.77% decrease in shoot length, while DS caused a 10.93% decrease over 0Cd + no drought (Fig. 1B). When both 6Cd and DS were combined, the shoot length decreased by 16.26% compared to the 0Cd + no drought. In GA3-BC1, the shoot length increased by 18.70% over control under 0Cd + no drought. When 6Cd stress was applied along with the GA3-BC1 treatment, the shoot length increased by 21.63% over the control. Similarly, under DS, the shoot length was increased by 23.92% where GA3-BC1 was added compared to control. At 6Cd + DS, GA3-BC1 treatment resulted in 26.54% increase in shoot length from control. Results showed that in GA3-BC2 the shoot length was enhanced i.e., 31.75% compared to the control under 0Cd + no drought. However, at 6Cd, treatment GA3-BC2 resulted in 37.64% improvement in shoot length than the control. Similarly, the shoot length under DS increased by 39.74% whereas GA3-BC2 was applied as treatment over control. Under 6Cd + DS, GA3-BC2 treatment resulted in 44.99% increase in shoot length than the control.

When subjected to 6Cd stress, the root length decreased to 4.39 cm, representing a decrease of 6.20% compared to the control (Fig. 1C). Similarly, under DS, the root length further decreased to 3.93 cm, indicating a decrease of 16.30% over the control. At 6Cd + DS, the root length decreased to 3.70 cm, resulting in a decrease of 20.81% over control. Under GA3-BC1 at 0Cd + no drought, the root length was increased to 7.03 cm, representing a significant increase (50.14%) from the control. At 6Cd stress addition of GA3-BC1 caused an enhancement of 50.49% over the control. In the case of DS, the root length was increased (52.16%) where GA3-BC1 was applied compared to the control. It was noted that treatment of GA3-BC1 under 6Cd + DS, exhibited a significant increase i.e., 42.84% from control. Results showed that GA3-BC2 under 0Cd + no drought caused an improvement of 82.76% than a control for root length. When GA3-BC2 was applied at 6Cd, the root length was increased to 86.83% increase over control. In the case of DS, the root length was enhanced, indicating a significant increase (95.76%) than the control. Results showed that 6Cd + DS, treatment GA3-BC2 led to an increase (99.73%) in root length over the control.

In control, the seedling length was 16.25 cm at 0Cd + no drought (Fig. 1D). Exposure to 6Cd resulted in a decrease of 6.72%, while DS caused a decrease of 12.40% in seedling length over control of 0Cd + no drought. The combination of 6Cd and DS further reduced the seedling length by 17.71% than the control of 0Cd + no drought. Under 0Cd + no drought, there was an increase of 27.75% in GA3-BC1 than control. In the case of 6Cd, GA3-BC1 caused an increase of 29.98% from control. Similarly, the seedling length increased by 31.72% under DS compared to control. AT 6Cd + DS, treatment GA3-BC1 resulted in an increase of 31.05% from control. For 0Cd + no drought, there was an increase of 46.44% observed where GA3-BC2 was applied than control. In 6Cd, the application of GA3-BC2 caused an increase of 51.87% over the control. Furthermore, under DS, the seedling length increased by 55.21% in comparison to the control. For 6Cd + DS, the addition of GA3-BC2 caused an enhancement of 60.13% in seedling length than control.

**Figure 2:**
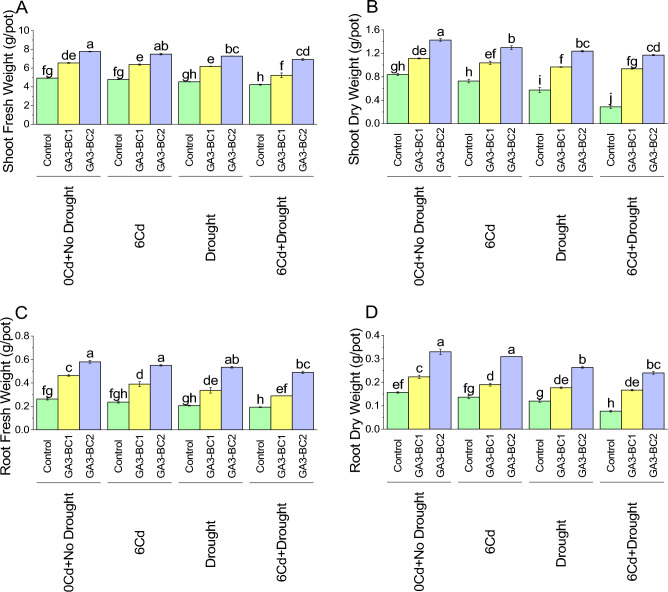
Impact of cadmium and drought stress on shoot fresh weight (**A**), shoot dry weight (**B**), root fresh weight (**C**), and root dry weight (**D**) of wheat amended with different levels of the GA3 enriched deashed biochar. The bars showed an average of three replicates, with standard error (SE). Statistical analysis using Tukey HSD revealed significant differences (*p* ≤ 0.05) between treatments, as indicated using different letterings on the bars.

In the control, under 0Cd + no drought, the shoot fresh weight was 4.92 g (Fig. 2A). When subjected to 6Cd, there was a decrease of 2.78%, while at DS, a decrease of 7.95% in shoot fresh weight compared with the control of 0Cd + no drought. When both 6Cd and DS were present, the shoot fresh weight was decreased by 14.01% over control. In the case of GA3-BC1, the shoot fresh weight was increased significantly i.e., 32.97% compared to the control at 0Cd + no drought. Under 6Cd, the GA3-BC1 treatment showed an increase of 33.15% than control. Similarly, under DS, the addition of GA3-BC1 treatment caused an increase in shoot fresh weight (36.42%) over the control. Similarly, applying GA3-BC2 treatment also significantly increased shoot fresh weight over the control at 0Cd + no drought. For GA3-BC2 at 0Cd + no drought, there was an increase of approximately 57.55% over control. At 6Cd, GA3-BC2 resulted in an increase of 56.34% than control. Furthermore, under DS in GA3-BC2 treatment, the shoot fresh weight was increased by 60.56% over the control. In the case of 6Cd + DS there was an increase (63.59%) observed in shoot fresh weight compared to the control where GA3-BC2 was applied.

In the control, under 0Cd + no drought, the shoot dry weight was 0.83 g (Fig. 2B). When subjected to 6Cd, there was a decrease of 13.20% in shoot dry weight, while DS resulted in a decrease of 31.45% over at 0Cd + no drought. The 6Cd + drought led to a significant decrease in shoot dry weight by 65.78% compared to the control of 0Cd + no drought. In GA3-BC1 at 0Cd + no drought, there was an increase of 33.07% compared to the control. When subjected to 6Cd, treatment GA3-BC1 showed an increase of 42.66%. Under DS, the shoot dry weight increased by 68.61%. The GA3-BC1 under 6Cd + drought resulted in an increase of 226.74% compared to the control. Treatment GA3-BC2 caused an increase of approximately 70.52% over control at 0Cd + no drought. In the case of 6Cd GA3-BC2 treatment increased to 78.44. At DS, the shoot dry weight increased by approximately 115.70% than the control. For 6Cd + drought, GA3-BC2 treatment resulted in an enhancement of 308.13% compared to the control.

Exposure to 6Cd stress resulted in a decrease to 0.23 g, while DS led to a further reduction to 0.20 g as compared to control at 0Cd + no drought for root fresh weight (Fig. 2C). The 6Cd + drought caused an additional decrease, with the root fresh weight reaching 0.19 g over control at 0Cd + no drought. At 0Cd + no drought treatment, GA3-BC1 caused root fresh weight to increase to 0.46 g, representing a significant increase 75.95% over control at 0Cd + no drought. In the case of 6Cd, application of GA3-BC1 treatment, the root fresh weight increased to 0.39 g, reflecting a 64.79% increase over the control. Similarly, under DS, the root fresh weight was increased up to 0.33 g, indicating a 62.90% enhancement as compared to the control. For 6Cd + drought, GA3-BC1 treatment resulted in 50.00% increase over control. Treatment GA3-BC2 at 0Cd + no drought resulted in significant improvement i.e., 120.26% of root fresh weight than control. It was noted that GA3-BC2 caused a significant increase (132.39%) over the control at 6Cd + drought. Furthermore, under DS, the root fresh weight showed a significant increase of 158.06% as compared to the control. The GA3-BC2 treatment under 6Cd + drought enhanced root fresh weight up to 153.45% than the control.

Exposure to 6Cd decreased root dry weight to 0.14 g, which represented a 12.72% decrease compared to the control of Cd + no drought (Fig. 2D). Similarly, under DS, the root dry weight decreased to 23.08% over control of 0Cd + no drought. The 6Cd + drought decreased root dry weight to 51.03% than the control of 0Cd + no drought. In treatment GA3-BC1, the root dry weight increased to 0.22 g, which represented a 42.55% increase compared to the control at 0Cd + no drought. When 6Cd stress was applied, GA3-BC1 caused an improvement in the root dry weight to 0.19 g, which was 39.02% increase compared to the control. Similarly, under DS, the root dry weight increased to 0.18 g, representing a 47.23% increase compared to the control. The GA3-BC1 treatment at 6Cd + drought resulted in a root dry weight of 0.17 g, which was a 117.39% increase compared to the control. Under 0Cd + no drought, treatment GA3-BC2 the root dry weight increased to 0.33 g, representing a 110.63% increase compared to the control. In 6Cd stress, the root dry weight increased 126.82% whereas GA3-BC2 was applied over control. Furthermore, under DS, the root dry weight increased to 0.26 g, which represented a 119.44% increase in control. In the case of 6Cd + drought, treatment GA3-BC2 resulted in 213.03% increase in root dry weight compared to the control.

**Figure 3:**
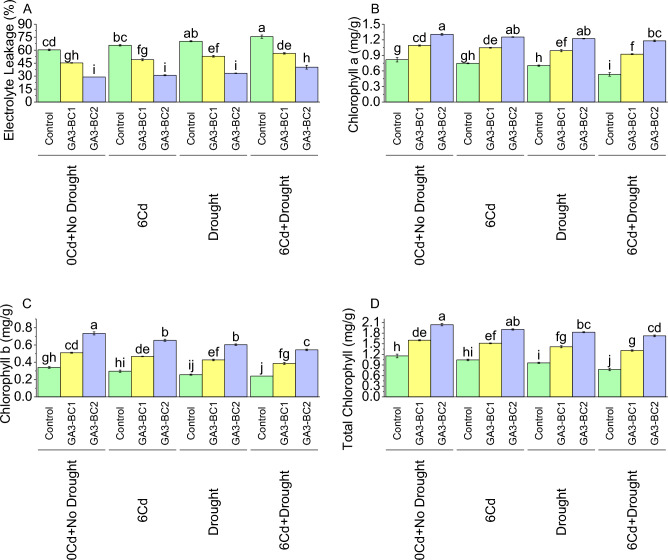
Impact of cadmium and drought stress on electrolyte leakage (**A**), chlorophyll a (**B**), chlorophyll b (**C**), and total chlorophyll (**D**) of wheat amended with different levels of the GA3 enriched deashed biochar. The bars showed an average of three replicates, with standard error (SE). Statistical analysis using Tukey HSD revealed significant differences (*p* ≤ 0.05) between treatments, as indicated using different letterings on the bars.

The application of the GA3-BC1 treatment led to a significant decrease of 24.86% in electrolyte leakage (EL) compared to the control at 0Cd + no drought (Fig. 3A). In the case of 6Cd, GA3-BC1 represents a 25.38% reduction in EL over control. Furthermore, the EL signified a 24.64% decrease from control under DS. Results showed that GA3-BC1 treatment at 6Cd + DS resulted in a 25.55% decline in EL compared to the control. It was noted that GA3-BC2 at 0Cd + no drought caused a significant 51.93% reduction in EL over control. When exposed to 6Cd, GA3-BC2 treatment caused a 52.79% decline in control. In DS, the EL reflects a 52.61% decrease over control. At 6Cd + DS, GA3-BC2 treatment resulted in a 46.70% decline compared to the control.

Under 0Cd + no drought, the control exhibited an average chlorophyll a content of 0.82 mg/g (Fig. 3B). Application of the GA3-BC1 treatment led to a significant increase in chlorophyll a, with a mean value of 1.09 mg/g, while GA3-BC2 treatment resulted in an even higher chlorophyll a content, with an average of 1.31 mg/g at 0Cd + no drought. When exposed to 6Cd, the control chlorophyll content was 0.75 mg/g. However, both GA3-BC1 and GA3-BC2 treatments exhibited a significant increase in chlorophyll a, with means of 1.05 mg/g and 1.25 mg/g, respectively at 6Cd. Under DS, the control displayed an average chlorophyll a content of 0.70 mg/g. The addition of GA3-BC1 and GA3-BC2 treatments caused enhancement in chlorophyll a to 0.99 mg/g and 1.22 mg/g, respectively under DS. In the case of 6Cd + Drought, the control showed the lowest chlorophyll a content, with an average of 0.53 mg/g. However, both GA3-BC1 and GA3-BC2 treatments significantly increased chlorophyll a at 6Cd + Drought, with means of 0.92 mg/g and 1.18 mg/g, respectively.

In 0Cd + no drought, the control displayed an average chlorophyll b content of 0.34 mg/g (Fig. 3C). The addition of GA3-BC1 resulted in an enhancement of chlorophyll b, with a mean value of 0.51 mg/g, while GA3-BC2 treatment showed a more significant increase, with an average content of 0.73 mg/g. In the case of 6Cd, the control resulted in a chlorophyll b content of 0.30 mg/g. However, both GA3-BC1 and GA3-BC2 treatments caused enhancement in chlorophyll b, providing means of 0.47 mg/g and 0.65 mg/g, respectively at 6Cd. In DS, the control showed chlorophyll b content of 0.26 mg/g. The addition of GA3-BC1 and GA3-BC2 treatments led to an enhancement in chlorophyll b i.e., 0.43 mg/g and 0.60 mg/g, respectively at DS. Results showed that in 6Cd + Drought, the control produced the lowest chlorophyll b content (0.24 mg/g). Nevertheless, both GA3-BC1 and GA3-BC2 treatments significantly improved chlorophyll b, resulting in means of 0.39 mg/g and 0.54 mg/g, respectively.

In control at 0Cd + no drought, the mean total chlorophyll value was 1.16 mg/g (Fig. 3D). When subjected to 6Cd, the total chlorophyll was declined to 1.04 mg/g. Similarly, in DS, the total chlorophyll further declined to 0.96 mg/g, The 6Cd + Drought resulted in a significant reduction in total chlorophyll, with a mean value of 0.77 mg/g compared to the control. Under 0Cd + no drought, the mean total chlorophyll was 1.60 mg/g, reflecting an increase of 38.33% compared to the control. Similarly, at 6Cd, the total chlorophyll was 1.51 mg/g, representing a 45.05% increase compared to the control. In the case of DS, the total chlorophyll mean further increased to 1.42 mg/g, indicating a 47.57% increase compared to the control. The 6Cd + Drought resulted in a total chlorophyll mean of 1.31 mg/g, demonstrating a significant increase of 69.40% compared to the control. In 0Cd + no drought, the mean total chlorophyll was 2.04 mg/g, showing a 76.37% increase compared to the control. Results showed that at 6Cd, the total chlorophyll was 1.91 mg/g, representing an 82.75% increase over control. Under DS, the total chlorophyll mean further increased to 1.83 mg/g, indicating a significant 90.28% increase compared to the control. The 6Cd + Drought caused improvement in total chlorophyll (1.72 mg/g), demonstrating a significant increase of 122.85% than control.

### 2,2-diphenyl-1-picrylhydrazyl (DPPH) activity, glutathione peroxidase (GPx), glutathione reductase (GR) and glutathione S-transferase (GST)

**Figure 4:**
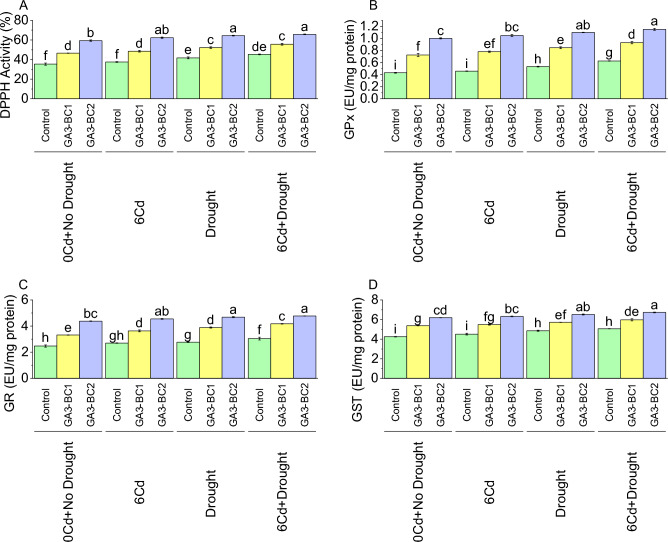
Impact of cadmium and drought stress on DPPH (**A**), GPx (**B**), GR (**C**) and GST (**D**) of wheat amended with different levels of the GA3 enriched deashed biochar. The bars showed an average of three replicates, with standard error (SE). Statistical analysis using Tukey HSD revealed significant differences (*p* ≤ 0.05) between treatments, as indicated using different letterings on the bars.

In the control under 0Cd + no drought, the mean DPPH activity was 35.35% (Fig. 4A). When treated with GA3-BC1, there was a noticeable increase of 31.63% in DPPH activity over the control at 0Cd + no drought. However, the most significant enhancement in DPPH activity was observed in the GA3-BC2 treated group, with a significant 67.80% increase from the control treatment at 0Cd + no drought. Moving on 6Cd exposure, the control showed a DPPH activity of 37.49%. Application of GA3-BC1 led to a 29.32% increase in DPPH activity in contrast to the control under 6Cd stress. Meanwhile, GA3-BC2 showed an even more significant improvement, with a significant 66.15% increase in DPPH activity over control under 6Cd stress. Under DS, the control exhibited a DPPH activity of 41.73%. When subjected to GA3-BC1 treatment over control, there was a 25.06% increase in DPPH activity under DS. Treatment GA3-BC2, on the other hand, related to the control, showed a more significant enhancement with a 54.45% increase in DPPH activity under DS. Furthermore, the 6Cd + DS showed a control DPPH activity of 45.29%. Treatment with GA3-BC1 resulted in a 22.84% increase DPPH activity than control. In comparison to the control under 6Cd + DS, treatment GA3-BC2 exhibited a significant 45.14% increase in DPPH activity.

In 0Cd + no drought, the control exhibited a mean glutathione peroxidase (GPX) activity of 0.43 EU/mg protein (Fig. 4B). However, when treated with the GA3-BC1 treatment compared to the control, there was a significant 68.32% increase in GPX activity 0Cd + DS. Similarly, GA3-BC2 treatment under 0Cd + DS resulted in 132.23% increase in GPX activity over the control. Under 6Cd, the control exhibited a GPX activity mean of 0.46 EU/mg protein. Treatment with GA3-BC1 increased GPX activity by 70.50% over the control under 6Cd. Meanwhile, GA3-BC2 treatment led to a significant 128.91% increase in GPX activity over the control at 6Cd. Under DS, the control showed a GPX activity mean of 0.53 EU/mg protein. As compared to the control under DS, GA3-BC1 treatment 58.72% increased GPX activity. Under DS, GA3-BC2 treatment resulted in a 106.13% increase in GPX activity over control. In the case of 6Cd + Drought, the control displayed a GPX activity mean of 0.63 EU/mg protein. Treatment with GA3-BC1 led to a 48.59% increase in GPX activity from the control under 6Cd + Drought. Meanwhile, GA3-BC2 treatment in 6Cd + Drought resulted in a 83.66% increase in GPX activity over the control.

The control showed an average GR activity of 2.47 EU/mg protein in 0Cd + no drought (Fig. 4C). When comparing the control under the 0Cd + no drought, GA3-BC1 caused a significant increase of 34.28% in GR activity, and this increase became even more pronounced at 77.09% when GA3-BC2 was applied over the control under 0Cd + no drought. A similar trend was observed under the 6Cd stress condition, with GA3-BC1 and GA3-BC2 treatments leading to respective percentage increases of 34.68% and 68.56% compared to the control. Under drought stress GA3-BC1 and GA3-BC2 treatments resulted in 40.41% increases and 69.15% increase in GR activity respectively, compared to the control. Moreover, the combination of 6Cd + Drought stress revealed significant changes, with GA3-BC1 and GA3-BC2 leading to 37.24% increases and 56.96%, respectively, when compared to the control under 6Cd and drought stress.

Under 0Cd + no drought, the control exhibited a GST level of 4.25 EU/mg protein (Fig. 4D). When treated with GA3-BC1, there was a significant increase of 26.34% in GST from the control at 0Cd + no drought. Similarly, GA3-BC2 treatment resulted in a more significant increment of 45.69% in GST at 0Cd + no drought compared to control. When exposed to 6Cd, the control showed a GST 4.51 EU/mg protein. The application of GA3-BC1 caused a rise of 21.64% than the control under 6Cd. Over control, GA3-BC2 treatment led to a more significant increase of 40.10% in GST level at 6Cd. Under DS, the control resulted in GST activity of 4.86 EU/mg protein. Treatment GA3-BC1 caused an increase in GST activity by 17.72% over the control under DS. Treatment GA3-BC2 under DS showed a higher increase of 34.21% in GST than the control. In 6Cd + DS, the control showed GST level of 5.06 EU/mg protein. Over the control under 6Cd + DS, GA3-BC1 led to a 17.93% increase in GST. In comparison to the control under 6Cd + DS, GA3-BC2 treatment caused a significant increase of 32.79% in GST level.

### Lipid peroxidation, superoxide dismutase (SOD), catalase (CAT) and ascorbate peroxidase (APx)

**Figure 5:**
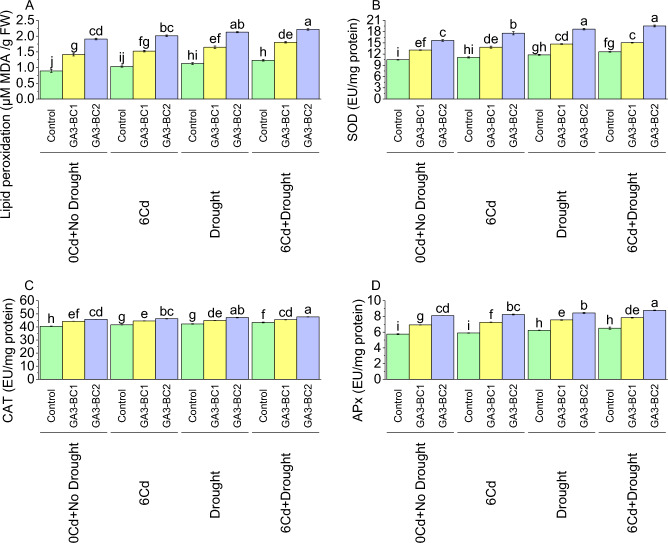
Impact of cadmium and drought stress on lipid peroxidation (**A**), SOD (**B**), CAT (**C**), and APx (**D**) of wheat amended with different levels of the GA3 enriched deashed biochar. The bars showed an average of three replicates, with standard error (SE). Statistical analysis using Tukey HSD revealed significant differences (*p* ≤ 0.05) between treatments, as indicated using different letterings on the bars.

The mean lipid peroxidation level in the control showed a of 0.89 µM MDA/g FW under 0Cd + no drought (Fig. 5A). However, when GA3-BC1 treatment was applied, there was a significant 58.16% increase in lipid peroxidation over the control under 0Cd + no drought. In the case of 0Cd + no drought, GA3-BC2 treatment resulted in a significant 113.41% increase in lipid peroxidation activity compared to the control. Under the 6Cd, the control showed a mean lipid peroxidation level of 1.03 µM MDA/g FW. When treated with GA3-BC1, there was a 47.34% increase in lipid peroxidation over control under 6Cd stress. Similarly, the application of GA3-BC2 caused in a significant increase (94.59%) in lipid peroxidation activity compared to control. For DS, the control exhibited a mean lipid peroxidation level of 1.13 µM MDA/g FW. The application of GA3-BC1 led to an increase (45.85%) in lipid peroxidation from the control at DS. Meanwhile, the GA3-BC2 treatment compared to the control indicated an increase i.e. of 88.37% in lipid peroxidation activity in DS. In 6Cd + Drought, the control had a mean lipid peroxidation level of 1.23 µM MDA/g FW. Application of GA3-BC1 treatment led to a 46.39% increase in lipid peroxidation over the control under 6Cd + Drought. Furthermore, the GA3-BC2 represents a significant 79.52% increase in lipid peroxidation activity compared to the control at 6Cd + Drought.

The mean superoxide dismutase (SOD) activity in control was recorded 10.50 EU/mg in 0Cd + no drought (Fig. 5B). When GA3-BC1 treatment was applied in 0Cd + no drought a significant increase (24.65%) in SOD activity was observed over control. Treatment GA3-BC2 resulted in a significant 49.16% enhancement in SOD activity than control under 0Cd + no drought. In case of 6Cd, the control showed SOD activity of 11.13 EU/mg protein. When exposed to GA3-BC1 treatment under 6Cd, there was a 24.55% increase in SOD activity from control. The GA3-BC2 treatment also showed significant increase, with a 58.08% enhancement in SOD activity compared to control under 6Cd. During DS, the control showed a mean SOD activity of 11.81 EU/mg protein. Treatment GA3-BC1 resulted in 24.41% increase in SOD activity compared to control at DS. Similarly, GA3-BC2 showed a significant increase (58.06%) in SOD activity compared to control under DS. For 6Cd + DS, the control had a mean SOD activity of 12.62 EU/mg protein. Treatment with GA3-BC1 led to a 19.37% increase in SOD activity than control under 6Cd + DS. Notably, the GA3-BC2 treatment caused a significant increase (54.83%) in SOD activity over control under 6Cd + DS.

For 0Cd + no drought, the control showed a catalase (CAT) activity of 10.50 EU/mg protein (Fig. 5C). The addition of GA3-BC1 caused a 24.65% increase in CAT activity over control under 0Cd + no drought. In addition, the GA3-BC2 at 0Cd + no drought led to a significant increase (49.16%) in CAT activity compared to the control. Exposure to 6Cd resulted in CAT activity of 11.13 EU/mg protein in control. Treatment with GA3-BC1 showed a 24.55% enhancement, while the GA3-BC2 showed a significant 58.08% improvement in CAT activity than control under 6Cd. Under DS, the control resulted in a CAT activity of 11.81 EU/mg protein. It was noted that GA3-BC1 treatment led to a 24.41% increase, while GA3-BC2 treatment showed a significant 58.06% increase in CAT activity than control at DS. For 6Cd + Drought, the control exhibited a CAT activity of 12.62 EU/mg protein. Treatment with GA3-BC1 resulted in a 19.37% increase and GA3-BC2 54.83% compared to control in 6Cd + Drought.

Results showed that at 0Cd + no drought, the control exhibited a mean APx level of 5.75 EU/mg protein (Fig. 5D). However, when treated with GA3-BC1 and GA3-BC2, there was a significant increase of 20.43% and 40.95%, respectively over the control under 0Cd + no drought. In 6Cd, the control had a slightly higher mean APx level of 5.90 EU/mg protein. In comparison to control, GA3-BC1 and GA3-BC2 resulted in increases of 23.11% and 39.84% respectively at 6Cd. Moving on to DS, the control exhibited a mean APx level of 6.23 EU/mg protein. Treatment with GA3-BC1 and GA3-BC2 led to percentage increases of 21.30% and 35.42% in contrast to the control under DS. The control showed a mean APx level of 6.51 EU/mg protein at 6Cd + Drought. In treatments GA3-BC1 and GA3-BC2, the APx levels increased by 20.53% and 34.74% from the control under 6Cd + Drought.

### Convex hull and hierarchical cluster plots

**Figure 6:**
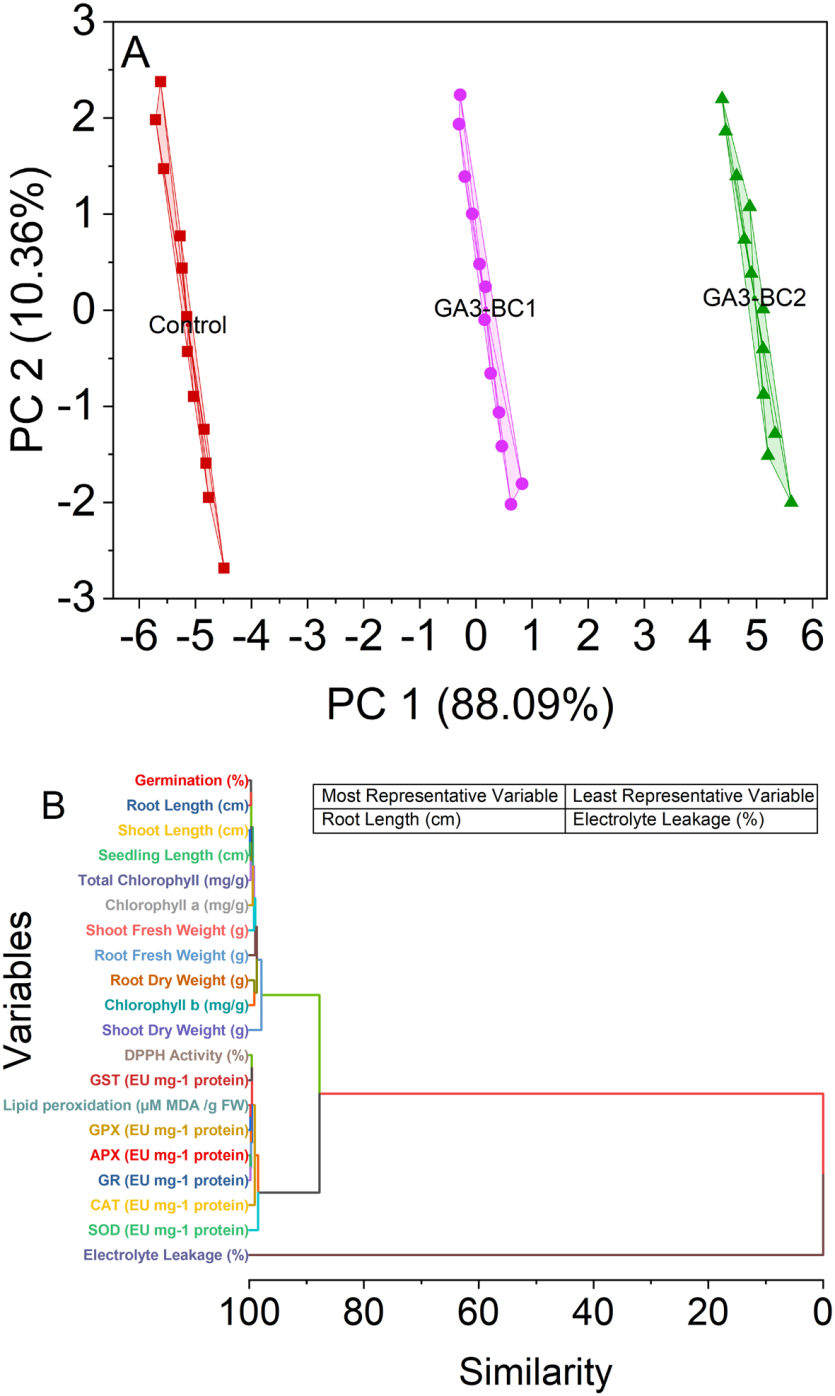
Convex hull cluster plot for treatments (**A**) and hierarchical cluster plots (**B**) for studied attributes.

For the control treatment group, the data points cluster around the negative PC1 axis, suggesting a distinct pattern in this dimension (Fig. 6A). This is reflected in the tight grouping of points along the PC1 axis, indicating a relatively consistent response within the Control. Meanwhile, the GA3-BC1 treatment group shows a dispersion of data points across both PC1 and PC2 axes, with some points extending into positive PC1 values. This indicates greater variability in the response within this treatment, potentially due to the influence of GA3-BC1. In contrast, the GA3-BC2 treatment group exhibits a spread of data points across both axes, with a concentration on positive PC2 values. This suggests a different response pattern than the other treatments, possibly influenced by GA3-BC2. The separation between treatment groups within the convex hull illustrates how these treatments impact the data differently, and it may imply distinct underlying mechanisms or effects.

The hierarchical cluster analysis results present pairwise similarity values between different pairs of variables. Notably, variables 3 and 4 exhibit a high similarity, with a similarity value of 0.12423, indicating that they share similar characteristics or patterns (Fig. 6B). Similarly, variables 14 and 15 also show a high similarity of 0.21785, suggesting a close relationship between them. Moving further, variables 36 and 38 have a remarkably high similarity value of 10.07057, indicating a very strong association between these two variables. Likewise, variables 37 and 38 exhibit a similarly high similarity value of 10.64562, further highlighting their close relationship. Variable 39 is part of a distinct cluster, having a similarity of 100 with itself, and this cluster includes all the variables. On the other hand, variables 8 and 32 have a relatively high similarity value of 1.07566, implying that they share some common characteristics. Variables 7 and 31 exhibit a significant similarity value of 0.84526, indicating a strong connection between them. Variables 11 and 36 have the highest similarity value in the dataset, at 2.15678, implying an exceptionally close relationship or shared patterns. Variables 9 and 10 show a similarity value of 0.90169, suggesting a significant similarity between these variables.

### Pearson correlation analysis

**Figure 7:**
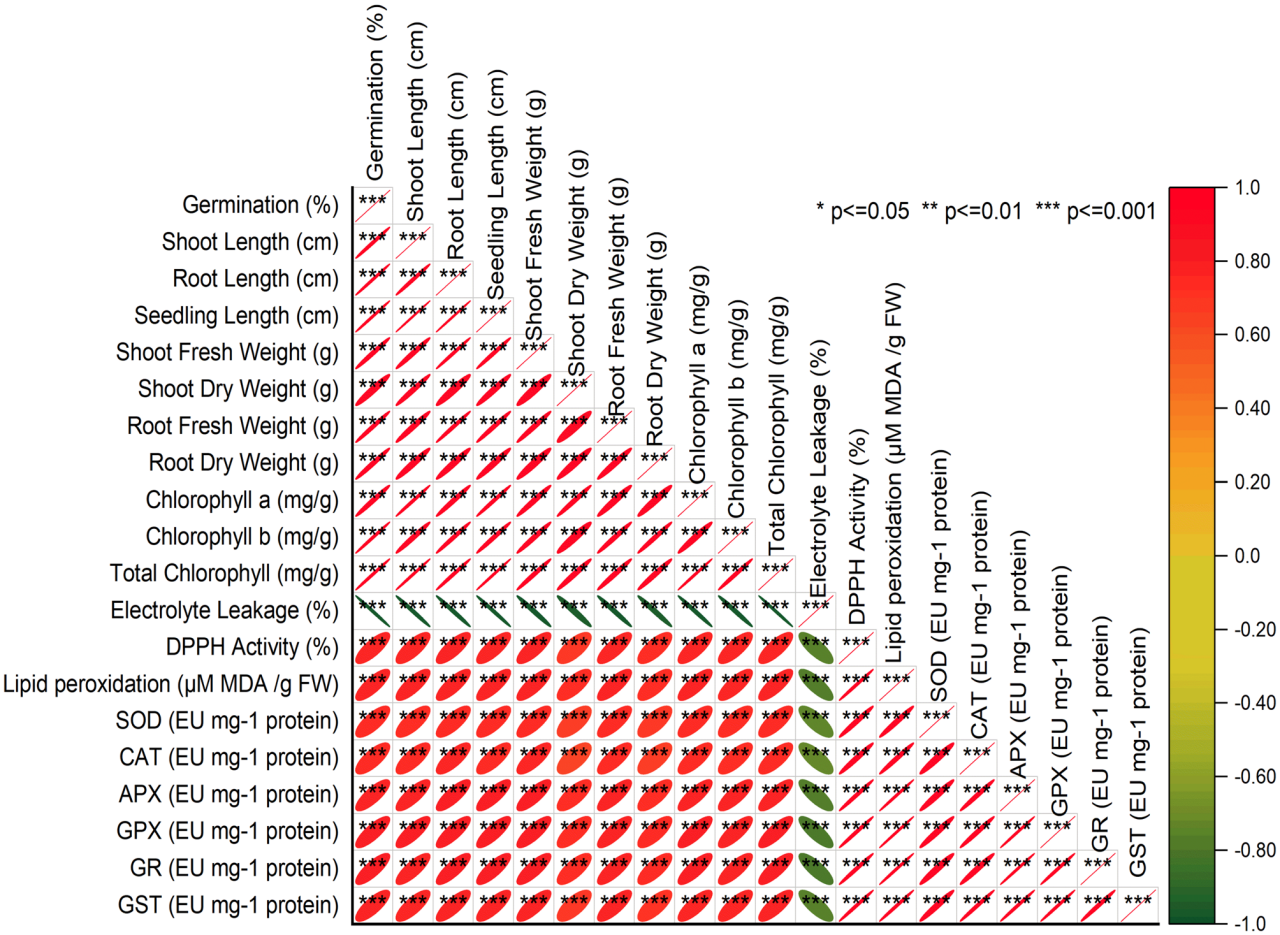
Pearson correlation analysis for the studied attributes.

Firstly, there was a robust positive correlation of 0.99085 between germination (%) and shoot length (cm), signifying that an increase in germination percentage is closely tied to a significant increase in shoot length in centimeters (Fig. 7). Similarly, the correlation between germination (%) and root length (cm) was also strongly positive at 0.99426, indicating that higher germination percentages are associated with longer root lengths. Furthermore, the relationship between shoot length (cm) and root length (cm) displayed an extremely high positive correlation of 0.98981, demonstrating a strong connection between these two variables. Longer shoots tend to coincide with longer roots. Moreover, a strong positive correlation of 0.99764 was observed between shoot length (cm) and seedling length (cm), indicating that increased shoot lengths correspond to longer seedling lengths. Additionally, the correlation between chlorophyll a (mg/g) and chlorophyll b (mg/g) was very strong at 0.99344, suggesting a highly related relationship between the concentrations of these two chlorophyll types, reflecting consistent chlorophyll composition. Total chlorophyll (mg/g) was found to be significantly correlated with chlorophyll a (mg/g) at a value of 0.99368, implying a close link between total chlorophyll concentration and the concentration of chlorophyll a. Moving on to physiological indicators, there was a significant negative correlation of -0.77738 between electrolyte leakage (%) and DPPH Activity (%), suggesting an inverse relationship between these two variables. Higher electrolyte leakage percentages were associated with lower DPPH activity percentages. Furthermore, lipid peroxidation (µM MDA/g FW) exhibited a strong positive correlation of 0.99285 with SOD (EU mg^-1^ protein), indicating that higher levels of lipid peroxidation are linked to increased levels of superoxide dismutase (SOD) activity. The correlation of 0.98225 between catalase (CAT) and ascorbate peroxidase (APX) activities signifies a strong positive relationship between these two enzymatic activities. Lastly, the association between glutathione peroxidase (GPX) and glutathione reductase (GR) activities showed a very high positive correlation of 0.99522, emphasizing a robust connection between these enzymatic activities. Similarly, the correlation of 0.99267 between glutathione S-transferase (GST) and glutathione peroxidase (GPX) activities underscores a strong positive relationship between these two critical enzymatic processes. These correlation results provide valuable insights into the interplay among these variables, aiding in a deeper understanding of the studied biological processes.

## Discussion

The combined effects of cadmium (Cd) exposure and drought stress exert significant impacts on various plant physiological and biochemical parameters^46^. Both Cd and drought stress negatively influence the germination process, root and shoot length, seedling length, as well as root and shoot fresh and dry weight. Cd accumulation in plants can lead to reduced germination rates, stunted root growth, and inhibited seedling development, while drought stress limits water availability for these processes^47^. The occurrence of both drought and Cd stress can result in the production of reactive oxygen species (ROS) within plant cells. Reactive oxygen species (ROS) encompass exceptionally reactive molecules like superoxide radicals, hydrogen peroxide, and hydroxyl radicals^48^. These ROS possess the potential to inflict harm upon cellular structures, including lipids, proteins, and DNA. Their reactivity can lead to detrimental consequences such as oxidative damage within the cells. Furthermore, Cd exposure and drought stress can disrupt chlorophyll synthesis and degrade chlorophyll molecules, resulting in reduced chlorophyll a, chlorophyll b, and total chlorophyll levels^49^. Additionally, both stressors induce cell membrane damage, indicated by increased electrolytic leakage (EL) levels.

The combined application of biochar and the plant growth regulator gibberellic acid (GA3) has shown significant effects on both plant physiological and biochemical properties, as well as soil health and fertility^50^. Biochar-GA3 treatment has been observed to enhance various plant physiological parameters, including seed germination, root and shoot growth, and seedling vigor^51^. It promotes nutrient uptake efficiency, improving plant nutrition and increased chlorophyll content. Additionally, biochar-GA3 application positively influences soil health by improving soil structure, enhancing water-holding capacity, and increasing soil aeration^52^. It also stimulates microbial activity, promoting nutrient cycling and availability. The high cation exchange capacity (CEC) of biochar aids in nutrient retention, reducing nutrient leaching, and enhancing nutrient management^53^. Furthermore, biochar-GA3 amendment helps alleviate soil acidity and reduce metal toxicity by acting as a pH buffer and absorbing heavy metals. Overall, the application of biochar-GA3 holds promise as a sustainable approach to enhance plant growth, improve soil quality, and support soil health and fertility, although further research is needed to understand the optimal application rates and long-term effects of this combination^54^.

The application of GA3 powder in conjunction with deashed biochar introduces various behaviors and interactions within the soil system. GA3 might be absorbed or adsorbed onto the surface of deashed biochar particles, with absorption involving the penetration of GA3 molecules into the porous structure of biochar and adsorption referring to the adherence of GA3 molecules onto the biochar’s surface^55^. Over time, GA3 gradually diffuses out of the biochar, making it available for uptake by plants. The presence of biochar offers stabilization to GA3, protecting it from degradation or rapid leaching in the soil. The porous structure of biochar serves as a reservoir, ensuring the proximity of GA3 to plant roots and its availability over an extended period. Additionally, biochar has the potential to enhance the bioavailability and uptake of GA3 by plants, improving its solubility and accessibility to plant roots^56^.

Furthermore, the application of the biochar-GA3 mixture can play a crucial role in cadmium (Cd) immobilization and drought stress alleviation in the soil. Biochar has been recognized for its ability to absorb heavy metals, including Cd, onto its surface^57^. When incorporated into Cd-contaminated soil, the biochar in the mixture acts as a sorbent, effectively binding with Cd ions and reducing their bioavailability. This immobilization process prevents Cd uptake by plants, minimizing Cd-induced toxicity and potential risks to human health through the food chain. In addition to its Cd immobilization properties, the biochar-GA3 mixture exhibits promising effects in alleviating drought stress^57^. Biochar enhances the soil's water-holding capacity and improves moisture retention, providing a buffer against water scarcity during drought periods. The porous structure of biochar acts as a reservoir, storing water and making it available to plant roots over an extended period. This mechanism aids in maintaining soil moisture levels, supporting plant growth, and mitigating the adverse effects of drought stress^58^.

## Conclusion

It is concluded that GA3-enriched biochar has the potential to mitigate the Cd and drought stress simultaneously when applied as an amendment in wheat crops. Application of GA3-BC2 showed more prominent results regarding improvements in growth attributes of wheat cultivated under Cd and drought stress. However, GA3-BC2 also showed better results for enchantment in the growth attributes and chlorophyll contents in wheat. Growers are recommended to use GA3-BC2 for the achievement of better wheat growth in Cd toxicity and drought conditions. More investigations are suggested at the field level to declare GA3-BC2 as the best treatment against drought and Cd stresses.

## Data Availability

All data generated or analysed during this study are included in this published article.
